# The Effects of Ovine-Derived Reinforced Tissue Matrix Surrounding Silicone-Based Implants in a Rat Prepectoral Reconstruction Model

**DOI:** 10.3390/bioengineering13020150

**Published:** 2026-01-28

**Authors:** Sai L. Pinni, Cameron Martin, Nicholas Fadell, Xiaochao Xia, Evan Marsh, Lauren Schellhardt, Xiaowei Li, Matthew D. Wood, Justin M. Sacks

**Affiliations:** Division of Plastic and Reconstructive Surgery, Department of Surgery, Washington University School of Medicine, St. Louis, MO 63110, USA; spinni@wustl.edu (S.L.P.); cameronm@wustl.edu (C.M.); xiaoweili@wustl.edu (X.L.)

**Keywords:** 3D printing, breast implant, capsule, foreign body response, rat model, silicone, acellular dermal matrix

## Abstract

Silicone-based implants have been widely used in breast reconstruction but have also been associated with poorly understood complications, including pathologic foreign body responses such as capsular contracture. In this study, we leveraged 3D-printing technology to generate silicone-based implants in a novel, anatomically relevant, prepectoral rat model. We used this model to evaluate the response to an extracellular matrix-based product: ovine-derived reinforced tissue matrix (RTM). Two-piece negative molds were developed through computer-aided design and 3D-printed. The molds were filled with various polydimethylsiloxane mixtures and dip-coated to fabricate implants. Implant material characterization revealed that the implants retained the original 3D-printed mold shape and qualitatively demonstrated a shell with an inner solid gel-like structure. Fabricated implants had smooth surfaces, as well as tunable features including implant stiffness (storage modulus). From initial studies in our rat model, placement of bilateral prepectoral implants allowed assessment of both muscle- and skin-facing capsules and were well-tolerated for at least 12 weeks. Comparison of the foreign body response between RTM-covered and uncovered (control) implants in this model revealed that the capsule thickness did not differ between groups at the 12-week endpoint. However, RTM reduced contractile fibroblasts (alpha-smooth muscle actin) and macrophages (Iba1) compared to the control. Our findings suggested that RTM may improve capsule quality by attenuating cells involved in fibrosis, even when total capsule thickness remains unchanged. However, these changes to cells involved in fibrosis were only observed at this early endpoint and may not predict long-term clinical outcomes.

## 1. Introduction

Breast augmentation is a common surgical procedure worldwide, with roughly a quarter million annual cases in the United States alone [1]. In addition to aesthetic reconstruction and revision of prior surgeries, indications for breast implant surgery also include oncologic reconstruction following mastectomy. Silicone is the most popular material for breast implants due to its low toxicity and relative physiologic inertness [2,3,4]. Despite their prevalence, silicone breast implants are not without potential complications.

The most common complication of implant-based breast surgery is capsular contracture [5]. In this process, the normal foreign body response of periprosthetic fibrotic encapsulation becomes pathologic, resulting in contractile remodeling and subsequent hardening around the implant [6,7]. This may eventually lead to pain, a distorted breast appearance, and the need for surgical correction via capsulectomy and capsulotomy [8,9,10,11,12]. Though the exact mechanism behind this transition from physiologic to pathologic inflammation remains unknown, the current literature suggests increased rates in capsular contracture with prepectoral implant placement, infection, hematoma/seroma, shear forces, and radiation [6,13,14]. Overall, studies have shown rates of up to 20% in breast augmentation and 30% in breast reconstruction [15,16].

The development of standardized pre-clinical models is crucial for investigating the sequelae of ongoing changes in clinical practice and their effects on mitigating adverse outcomes. Rodent models have been used previously to examine the exact mechanisms that underlie the foreign body response leading to capsular formation, as well as pathologic contracture [17,18,19,20]. Additionally, the recent use of 3D printing to form reproducible silicone implants has been implemented [21]. Among other animal models of foreign body response and capsular formation, such as pigs and rabbits, rats have been regarded as the one of the most affordable and reproducible with histologic findings akin to humans [22]. More specifically, samples of fibrous capsules from rat models of silicone implants have demonstrated similar histologic characteristics to human samples, including capsule thickening, monocyte and macrophage infiltration, and myofibroblast proliferation [20,22,23,24,25,26,27].

The management of capsular contracture has evolved over decades, beginning with open capsulotomy and progressing to total capsulectomy. Although the superiority of total capsulectomy over capsulotomy remains debated, total capsulectomy with implant exchange and site change is still regarded as the gold standard [28,29]. Against this backdrop, acellular dermal matrix (ADM) has also emerged as a promising adjunct in implant-based breast reconstruction [3]. ADMs are bioengineered scaffolds derived from human or animal tissue that can modulate the host’s foreign body response to implants and reduce complications such as capsular contracture [30,31]. A systematic review and meta-analysis of almost 500 total breast surgeries using ADM found capsular contracture rates of 0–6.6% and overall complication rates of 0–6.25% [32]. Similarly, a 13-year review of over 1500 breast reconstructions with ADM found a cumulative incidence rate of 0.8% and 1.9% incidence in irradiated breasts [33]. All incidences of capsular contracture occurred within the first two years of follow-up, with no increase in incidence with longer follow-up, suggesting that ADM does not simply delay occurrence but may actually prevent the development of capsular contracture [33].

The mechanisms behind this phenomenon have been further elucidated through histologic studies, which have found that ADM is associated with decreased myofibroblast presence, reduced inflammation, and less vascular proliferation within the periprosthetic capsule compared to implants without ADM coverage [34,35,36,37]. These findings suggest that ADM may alter the foreign body response through immunomodulatory and antifibrotic mechanisms, potentially mediated by reduced collagen, macrophage infiltration, and contractile fibroblasts, as well as the downregulation of profibrotic signaling pathways, such as transforming growth factor β1 (TGF-β1) [35]. Histologic scoring of capsule biopsies has even been correlated with 6-month postoperative Baker grade, which is often used for capsular contracture, further highlighting the utility of histologic data [38]. While the protective effects of ADM in general are increasingly recognized in clinical settings, preclinical models evaluating specific immune and fibrotic markers under controlled conditions may further reveal the mechanism’s underlying benefits.

While this model of experimentation has proven usable to study implant-associated pathologies, experimental designs are limited by the finite number of implant designs produced by commercial entities and the inherent lack of customizability present when using commercially made products. In this study, we leveraged 3D-printing technology to generate silicone-based implants in a novel, anatomically relevant, prepectoral rat model. Utilizing computer-aided design and 3D-printing technology, our method produced silicone implants for use in animal studies to enable evaluation of extracellular matrix-based products in rats. We evaluated the host response to a commercially available, ovine-derived reinforced tissue matrix (RTM), which is a bioscaffold derived from ovine extracellular matrix [21,39] and is similar to ADM. We characterized the effects of this RTM on capsular formation, fibrosis and inflammation markers, and neoplastic growth markers in this rat model, which reflected the normal foreign body response as opposed to pathological capsular contracture, as a means to understand RTM’s utility in silicone-based implant surgery.

## 2. Materials and Methods

### 2.1. Implant Mold Design, Fabrication, and Generation

**Figure 1:**
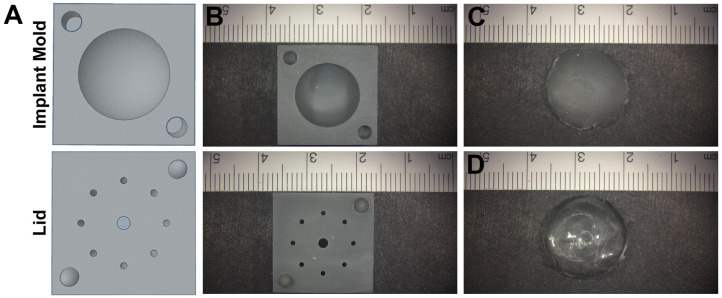
Implant design and fabrication. Hemispherical negative molds (“Implant Mold”) and mold covers, to facilitate reproducible implant cores, were constructed using computer-assisted design (**A**). Printed molds were used to contain silicone for curing to fabricate the implant core (**B**). Implant cores prior to dip-coating (**C**) and after dip-coating (**D**) had minimal differences in gross size. Scale bar (cm) shown.

Two-piece negative molds (Figure 1) were developed through computer-aided design with TinkerCAD (Autodesk, San Francisco, CA, USA), an open-source online 3D-design platform. The bottom mold (“Implant Mold”) contained a negative hemispherical imprint of the implant, while the mold cover (“Lid”) contained airholes to allow even curing of the silicone with protruding nodes to secure the two pieces together. The molds were 3D printed using stereolithography of Grey Photoreactive Resin (Formlabs, Somerville, MA, USA), a methacrylic acid ester-based product, on a Formlabs 3B+ printer (Formlabs). The molds were printed utilizing adaptive layering, resulting in molds with a layer resolution of up to 0.025 mm. This allowed for reduced print duration and a smooth, ridge-free surface. Mold dimensions used in this study to accommodate female Lewis rats (described in more detail later) had a hemisphere radius of 6.55 mm, resulting in a volume of 589 mm^3^ and a surface area of 404 mm^2^.

Upon completion of the 3D-printing cycle, the negative injection molds were placed in a Form Wash (Formlabs), a wash-bath containing 3 L of 70% Isopropyl alcohol, where excess residual resin from the printing process was removed. Molds were then left to dry for 10 min before the supportive scaffolding surrounding them was removed. Finally, the molds were placed in a FormCure (Formlabs) curing device set at 60 °C with 405 nm light exposure for 30 min.

To fabricate the silicone implants, polydimethylsiloxane (Slygard 184, Dow Corning, Midland, Mich) was mixed at a 50:1 silicone base-to-cure ratio and poured into the molds enclosed by the covers, where this mixture served as the “Core” of the implant. To generate “Stiff” implants, implant cores were allowed to cure for 48 h at room temperature, while softer implants (“Soft”) were generated by curing implant cores for 48 h at 60 °C. After the 48 h, implant cores were explanted from the molds and dip-coated in a different silicone mixture, now at a 1:1 base-to-cure ratio, at two 24 h intervals to serve as the “surface” or shell of the implant, similar to commercial silicone-based breast implants. After this shell process, implants were left in a water bath (~500 mL) for at least 7 days to wash residual chemicals. Before implantation in vivo, implants were dry heat sterilized for 48 h at 110 °C in autoclave pouches.

### 2.2. Implant Mechanical Characterization of Stiffness

Implants, fabricated as described, were formed and punched into 8 mm rounds at the center of the implant. The storage modulus of each implant was measured via oscillatory strain sweep on a HR-20 rheometer (TA Instruments, New Castle, DE, USA), using an 8 mm parallel plate configuration, a strain of 0.1–100%, and frequency of 1 Hz (n = 4 for each implant type). The results at a strain range of 0.1–10% was within the linear viscoelastic region for all implants tested.

### 2.3. Scanning Electron Microscopy (SEM) of Implants

The surface topography/texture of implants was evaluated with SEM. Samples were dehydrated in a graded ethanol series (10%, 30%, 50%, 70%, 90%, and 100% × 4) for 10 min each step. Following dehydration, the samples were cut with a double-sided razor blade or frozen in liquid nitrogen (LN2), and freeze cracked with a single-edge razor blade to expose cross-section views. Samples were loaded into a critical point drier (Leica EM CPD 300, Vienna, Austria) which was set to perform 12 CO_2_ exchanges at the slowest speed. Once dried, the samples were mounted, cross-section-side up, on 12 mm aluminum pin mounts with carbon adhesive tabs and coated with 10 nm of carbon and 6 nm of iridium (Leica ACE 600, Vienna, Austria). SEM images were acquired on a FE-SEM (Zeiss Merlin, Oberkochen, Germany) at 3.0 kV and 300 pA.

### 2.4. In Vivo Rodent Model and Tissue Harvest

Commercially available adult female Lewis rats (200 g, Charles River Laboratories, Wilmington, MA, USA) were utilized in this experiment. Surgical procedures and peri-operative care measures were conducted in compliance with the AAALAC accredited Washington University Institutional Animal Care and Use Committee (IACUC) and the National Institutes of Health guidelines. All animals were housed in a central animal care facility and provided with food (PicoLab rodent diet 20, Purina Mills Nutrition International, St. Louis, MO, USA) and water ad libitum. For the experimental design, two (2) hemispherical silicone breast implant prototypes were inserted bilaterally into the rat in the prepectoral plane. For studies involving RTM surrounding the implant, the left-sided implant was completely wrapped in 2-layer OviTex PRS (OviTex^®^ PRS by TELA Bio, Malvern, PA, USA) using a purse-string suture technique before implantation. Both implants were harvested at a 12-week endpoint for histological evaluation.

Implants and surgical instruments were autoclaved preoperatively for sterilization. Surgical procedures were performed under aseptic conditions without an operating microscope. Rats were anesthetized using a cocktail of ketamine (75 mg/kg; Fort Dodge Animal Health, Fort Dodge, IA, USA) and dexmedetomidine (0.5 mg/kg; Pfizer Animal Health, Exton, PA, USA). Animals did not receive preoperative antibiotics. The ventral surface of the animal was shaved and prepped with betadine and ethanol. Two 1 cm horizontal skin incisions were made at the inferior aspects of the pectoralis major muscles. Blunt dissection was performed to create a subcutaneous pocket. Implant pockets were irrigated thoroughly with sterile saline before and after insertion of the silicone implants. The skin incisions were closed with interrupted 4-0 nylon sutures. Anesthesia was reversed using atipamezole solution (0.1 mg/kg; Zoetis, Florham Park, NJ, USA). No postoperative antibiotics were administered, and no protective dressings were used. The animal recovered on a heating pad and was monitored for postoperative complications before returning to a central animal care facility. Sutures were removed on day 7 following the operation. Postoperative management included daily monitoring for signs of infection and/or distress and administering Buprenorphine SR™ (0.05 mg/kg; ZooPharm, Windsor, CO, USA) for postoperative pain. At the 12-week endpoint, animals were euthanized with an intraperitoneal administration of pentobarbital (150 mg/kg) and tissue harvested using the same described approach.

### 2.5. Histological Evaluation

Implants were harvested en bloc with up to 5 mm of surrounding skin and soft tissue, submerged in 10% neutral buffered formalin for 48 h, and dehydrated in 70% ethanol for 24 h. Fixed tissue units were sharply bisected, implants were removed, and the capsular tissue was dehydrated, processed, and embedded in paraffin. Samples were sectioned at 10 µm thickness by the Washington University Musculoskeletal Histology Core.

Colorimetric immunohistochemistry staining was performed by the Histology Core. Hematoxylin and eosin (H&E) staining was used to identify the capsule area surrounding the implant. Picrosirius red was used to determine the collagen density within the capsule area. Slides were imaged using a BS-2081 brightfield microscope (BestScope, Beijing, China) with a Sony Exmor IMX-264 camera and Toupview acquisition software (ToupTek Photonics Co. Ltd., Zhejiang, China). Fields were captured at 100× (10× objective) overall magnification, which represented 6–9% of the specimen. For quantifying capsule thickness in H&E fields, ImageJ with Fiji 2.16.0 was used by a blinded observer to take 5 measurements of capsule thickness per field, with 3 fields analyzed from both sides of the capsule (skin and muscle) (6 fields total for each specimen ranging from 36 to 54% of the capsule), for each animal. ImageJ with Fiji was also used to capture (using a blinded observer) and measure collagen density in picrosirius red fields. Images were converted to 8 bit, the intensity of the capsule area was measured, and then the intensity of the background (unstained area of the image) was subtracted from the final value. Collagen density was measured from 3 representative fields on each side of the implant (skin and muscle) for each animal. The periprosthetic capsule was defined as the collaged fiber layer of tissue starting at the implant-tissue interface and ending at the transition to underlying tissue. Qualitative assessments of collagen organization and density were also performed.

For fluorescent immunohistochemistry, paraffin sections were rehydrated, and antigen retrieval was performed by submerging slides in antigen retrieval reagent (R&D Systems) at 95 °C for 8 min and then rinsing in PBS. Sections were blocked for 1 h at room temperature using a solution of 8% normal goat serum, 2% bovine serum albumin, and 0.1% Triton X-100 diluted in PBS. After blocking, a solution containing the primary antibodies diluted in the blocking buffer was applied to the sections and incubated overnight at 4 °C. Primary antibodies were used to stain for pan macrophages (Iba1), pro-inflammatory or “M1” macrophages (CD86), anti-inflammatory or “M2” macrophages (CD206), contractile fibroblasts (alpha-smooth muscle actin [α-SMA]), profibrotic signaling pathway protein TGF-β1, and indications of metaplasia including cell proliferation (Ki-67), as well as proteins vimentin and cytokeratin (cytokeratin-20). Primary antibodies were followed by a PBS wash and then stained for the appropriate fluorochrome-conjugated secondary antibodies for 1 h at room temperature. The slides were then washed in PBS and sections were mounted with Fluorshield mounting medium with DAPI (Abcam) and imaged using a FV1000 confocal microscope and acquisition system (Olympus). Fields were captured at 600× (60× oil immersion objective) overall magnification for quantification. ImageJ with Fiji 2.16.0 was used to quantify the number of positive cells within each field. Five representative fields were analyzed per capsule tissue region (skin and muscle) for each animal.

### 2.6. Statistics

Statistical analysis was performed using GraphPad Prism version 9.5.0 (GraphPad Software, LLC, Boston, MA, USA). Each animal and its implant represented an ‘n’ value. We anticipated that a sample size of n = 10 per group would be sufficient power to detect an effect size of 0.25, at 80% power, α = 0.05, and standard deviation (SD) of 0.20). All data were represented as mean ± SD. Data sets were tested for normality using the Shapiro–Wilk test. Student’s *t* test was performed for comparison between 2 groups with Gaussian distribution. To assess the independent and combined effects of the implant group (control vs. RTM) and the tissue type (skin vs. muscle) on histologic outcomes, two-way ANOVA tests were performed for various outcomes: capsule thickness, collagen density, and percentage of cells. Post hoc analysis to directly compare groups was performed using Tukey’s multiple comparisons test. A significance level of *p* < 0.05 was used.

## 3. Results

### 3.1. Implant Characterization and Mechanical Properties

Before the evaluation of RTM, the silicone implant’s material properties were characterized. Implants, regardless of their perceived stiffness or dip-coating, retained the original shape of the 3D-printed molds (Figure 1). Implants constructed using a single-dip vs. double-dip coat in a high base-to-cure ratio of silicone, which generated a stiff yet thin silicone layer to mimic the shell of an implant, were not grossly different in appearance. Therefore, all implants were assessed to understand their basic mechanical properties.

**Figure 2:**
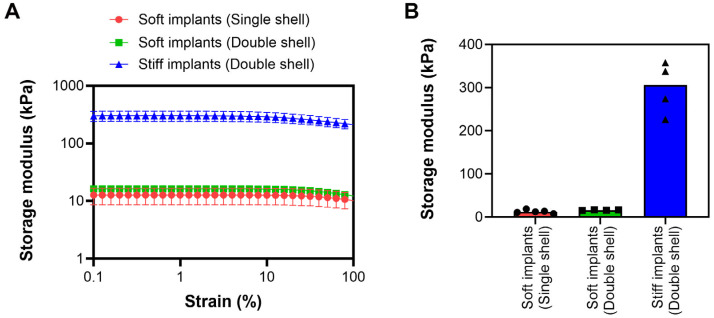
Mechanical characterization of implants using rheology. Implant stiffness was measured, which included storage modulus as a function of strain rate (**A**) and average storage modulus at a range of 0.1–10% strain (**B**). Data represented as mean ± standard deviation (SD) (n = 4/group).

Rheological testing was utilized to determine the mechanical properties of the implants where storage modulus (i.e., material stiffness) was specifically assessed. All implants were compressed within parallel plates at a variable strain rate. Compression at a variable strain rate revealed significant differences in storage modulus for the Soft vs. Stiff implants (Figure 2A). Implants fabricated to be less rigid (“Soft” implants) were less mechanically stiff compared to “Stiff” implants, regardless of their dip-coat quantity, as expected. Compression at the linear viscoelastic range of strain demonstrated Soft implants fabricated with a single or double dip-coat were not different from one another (Storage modulus: 12.6 ± 4.02 kPa vs. 16.2 ± 0.519 kPa, respectively; Figure 2B). Implants fabricated to be more rigid (“Stiff” implants) exhibited a storage modulus of 299 ± 60.3 kPa at the linear viscoelastic range of strain.

### 3.2. Implant Characterization and Surface Visualization

**Figure 3:**
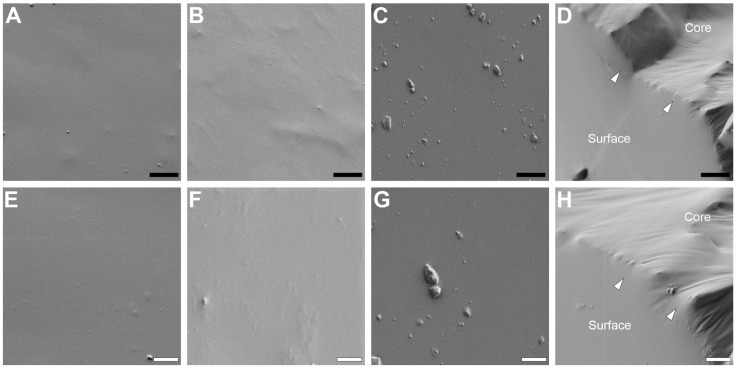
Scanning electron microscopy (SEM) of implant surfaces. SEM images of the soft single-coated implants (**A**,**E**), double-coated implants (**B**,**F**), and stiff double-coated implants demonstrating minimal texture (**C**,**G**). Implants that were freeze cracked to examine their interior core, regardless of mechanical properties (stiff double-coated implant shown), demonstrated no clear contrast between where the silicone dip-coated surface (“Surface”) interfaced (white arrows) with the silicone implant core (“Core”) (**D**,**H**). Black scale bars are 10 µm (**A**–**D**) and white scale bars are 4 µm (**E**–**H**).

Implants were assessed for surface characteristics using SEM, which qualitatively demonstrated similar surface topography between implants despite differences in mechanical properties (Figure 3). Implants were also freeze cracked to examine their interior core. Regardless of their mechanical properties (Stiff, double-coated implant shown in Figure 3D,H), implants demonstrated no clear contrast between where the silicone dip-coated surface interfaced with the silicone implant core. These results suggest that implants with variable mechanical properties can be fabricated with generally similar surface properties using the developed fabrication.

### 3.3. Baseline Observations of Foreign Body Response in the Prepectoral Model

**Figure 4:**
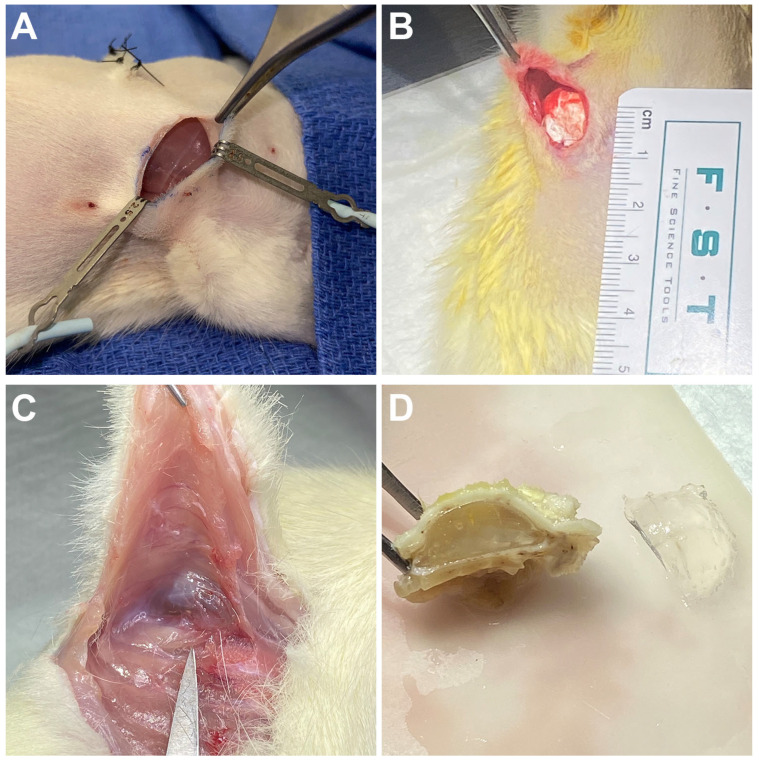
Implantation and harvest of stiff silicone implants in rats. Implants were inserted bilaterally using horizontal skin incisions at the inferior aspects of the pectoralis major muscles (**A**,**B**). After 3 months, the implants and surrounding tissue were harvested en bloc to retain the structure of the capsule and surrounding tissue for histological analysis (**C**,**D**).

A pilot study was utilized to generate basic histological characterization of stiff implants, evaluated in the novel prepectoral rat reconstruction model, to determine the degree of the foreign body response to the implants (Figure 4). Postoperatively, animals did not exhibit any signs of distress or require pain medicine. There were no noticeable deficits in function (i.e., feeding, grooming) or mobility/gait. Surgical sites healed well with no signs of infection, dehiscence, or wound-site mutilation. By the 12-week endpoint, some implants changed orientation, but all remained in their respective pectoral pocket without migration.

**Figure 5:**
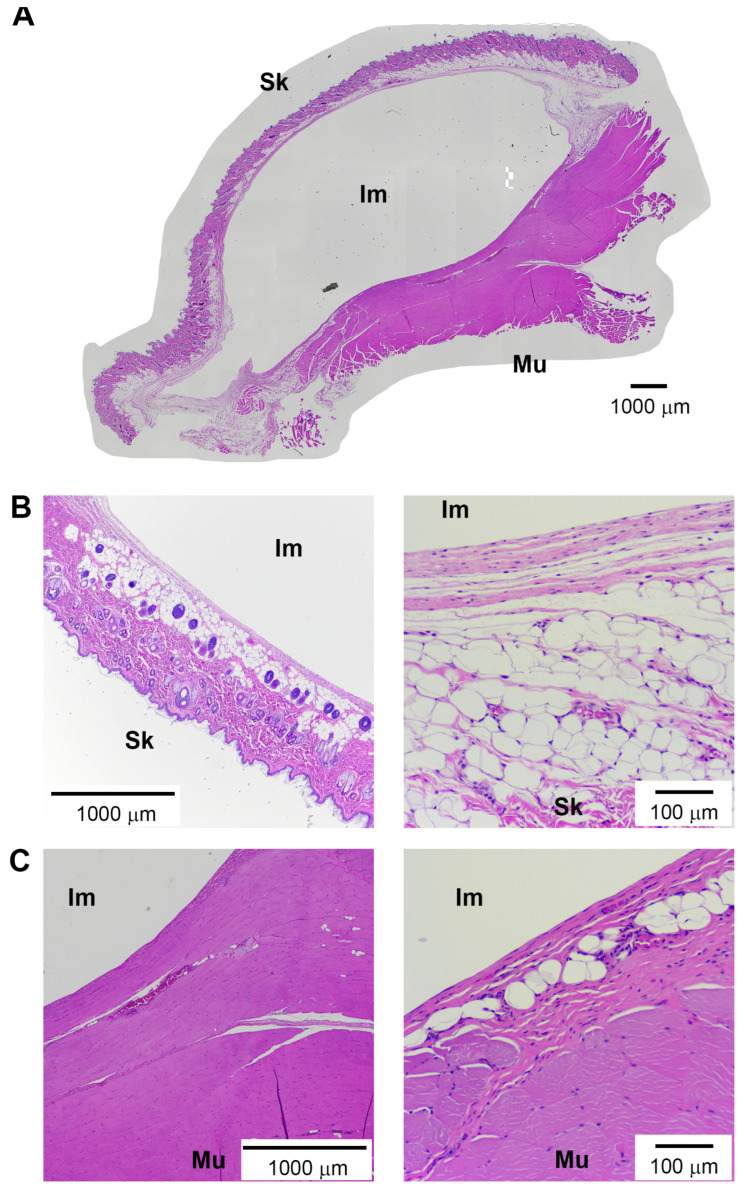
Histologic overview of breast implant capsule formation in the novel rat model. Representative images of an implant capsule from hematoxylin and eosin (H&E) staining. A complete view of the tissue surrounding the implant demonstrated a thin capsule surrounding the skin and muscle borders (**A**). Enhanced magnification revealed the capsule forming at the border of the implant (now removed) and skin (**B**) or muscle (**C**). Scale bars as indicated in each panel. “Im” indicates implant, “Sk” indicates skin, and “Mu” indicates muscle.

Gross observations of harvested implants showed no evidence of infection, seroma, hematoma, or disruption to surrounding anatomy. Harvested implants showed no noticeable change in volume or size. Implant surfaces remained smooth without any deficits or erosion. On visual examination of the bisected en bloc harvested implants, all expected tissue layers were seen including capsule formation around the implants (Figure 4C). This capsule was clearly distinguishable from the surrounding tissue, presenting at the border of the implant and either the surrounding skin or muscle (Figure 5). The implant capsule demonstrated lamellar collagen architecture with increased inter-fiber space and cellularity compared to the surrounding tissue.

### 3.4. Evaluation of RTM-Wrapped Implants for Capsule Formation

**Figure 6:**
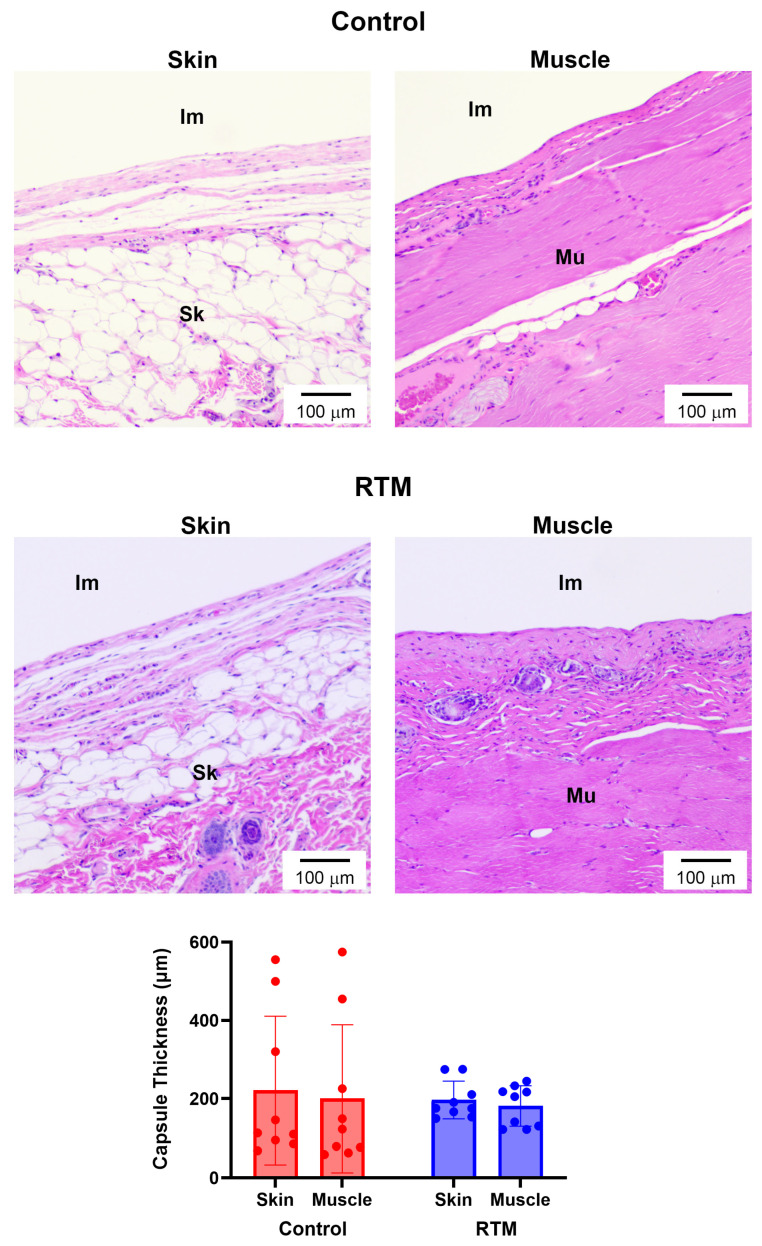
RTM coverage does not affect breast implant capsule thickness. Representative H&E-stained sections at 10× magnification comparing capsules formed around control and RTM-wrapped implants at the skin and muscle surfaces at 12 weeks. Scale bars = 100 µm. “Im” indicates implant, “Sk” indicates skin, and “Mu” indicates muscle. Quantification of capsule thickness (µm) is shown at the bottom. Data represented as mean ± SD (n = 10/group).

Based upon validation of the foreign body response in this model, RTM-wrapped implants were compared to unwrapped (control) implants at a 12-week endpoint. Through H&E staining to visualize the capsules, when accounting for capsule formation at the skin and muscle surfaces, there was no significant difference in average capsule thickness between the two implant groups (control: 211 ± 183 µm [range 66–537 µm] vs. RTM: 189 ± 49 µm [range 158–260 µm], *p* = 0.70; Figure 6). However, the capsule thickness range was four times larger for the control group compared to the RTM group. The main effects of the implant group (*p* = 0.64) and the tissue type (*p* = 0.70) were not significant.

### 3.5. Evaluation of RTM-Wrapped Implants for Fibrosis

**Figure 7:**
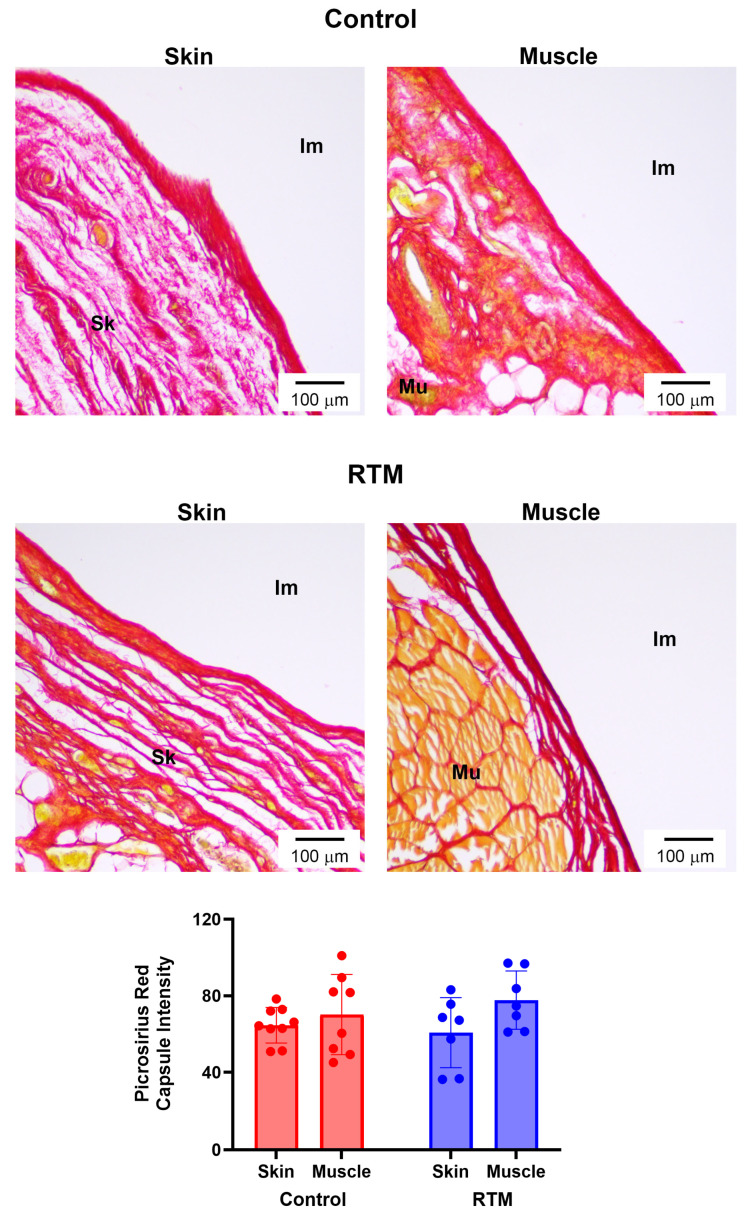
RTM coverage does not affect breast implant collagen density. Representative picrosirius red-stained sections at 10× magnification comparing capsules formed around control and RTM-wrapped implants at the skin and muscle surfaces at 12 weeks. Scale bars = 100 µm. “Im” indicates implant, “Sk” indicates skin, and “Mu” indicates muscle. Quantification of collagen density is shown at the bottom. Data represented as mean ± SD (n = 10/group).

**Figure 8:**
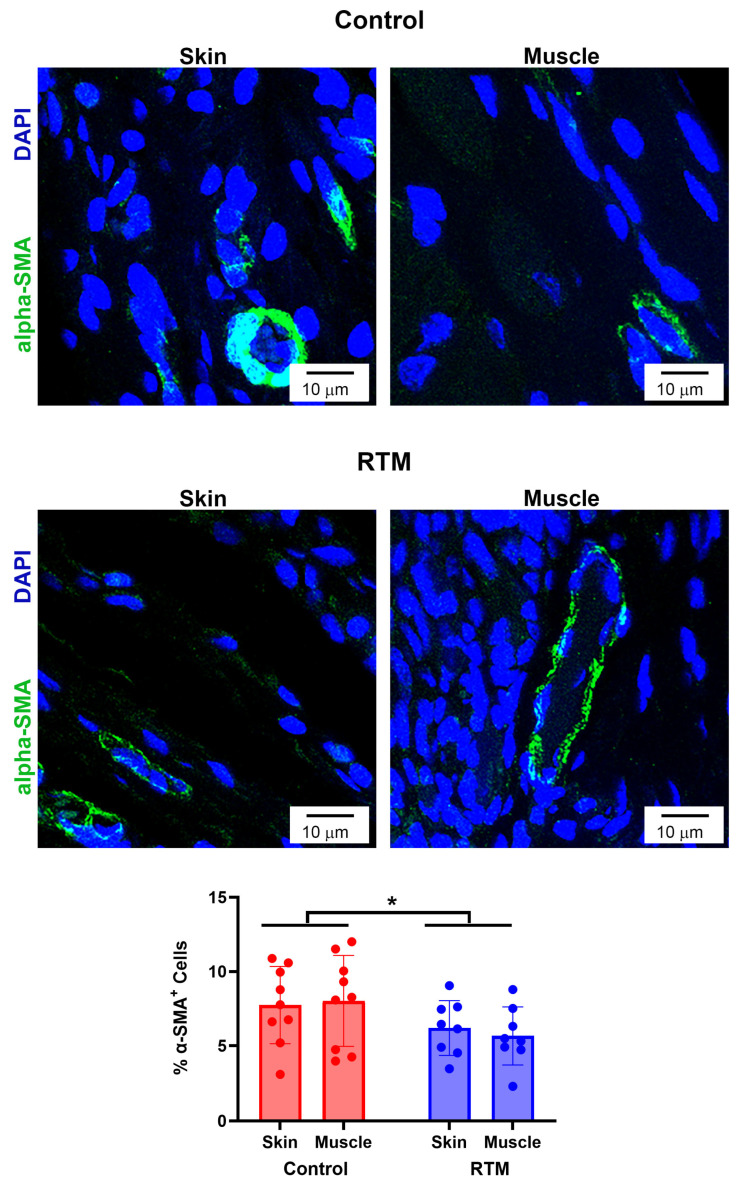
RTM coverage reduces contractile fibroblasts. Representative immunofluorescence staining for alpha-smooth muscle actin (α-SMA) at 60× magnification comparing capsules from control and RTM-wrapped implants at the skin and muscle surfaces. Scale bars = 10 μm. Quantification of the percentage of α-SMA–positive cells are shown at the bottom. Data represented as mean ± SD (n = 10/group). * indicates *p* < 0.05.

**Figure 9:**
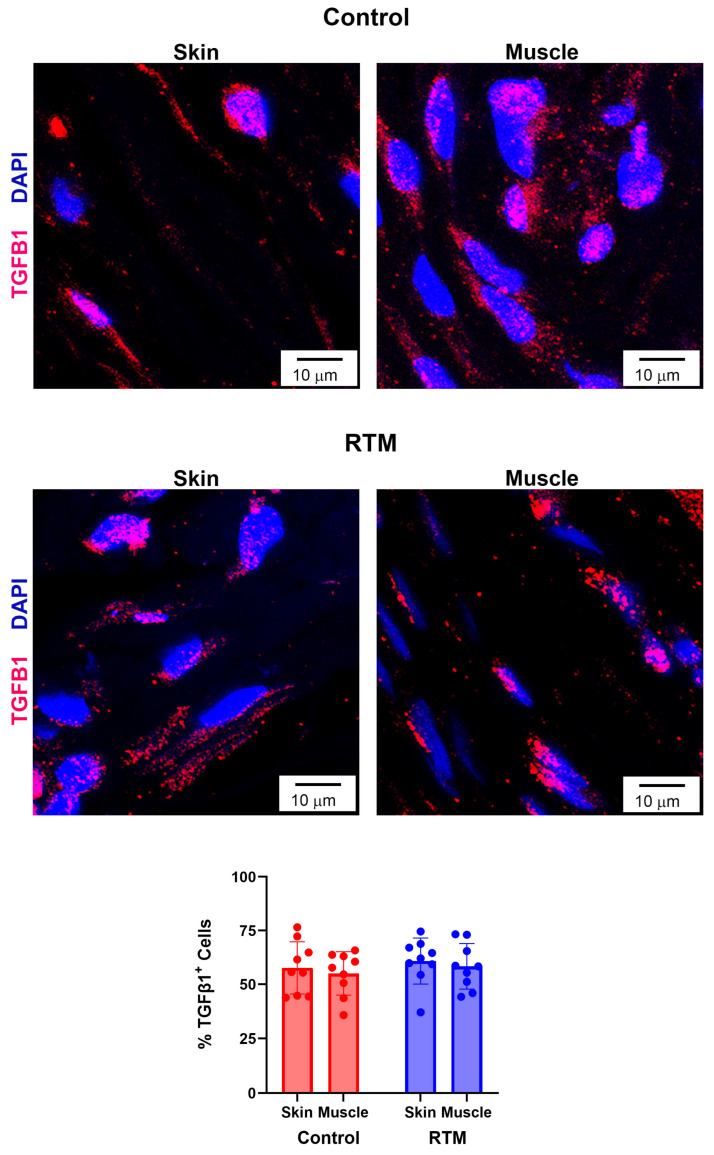
RTM coverage does not affect TGF-β1 expression. Representative immunofluorescence staining for TGF-β1 at 60× magnification comparing capsules from control and RTM-wrapped implants at the skin and muscle surfaces. Scale bars = 10 μm. Quantification of the percentage of TGF-β1–positive cells are shown at the bottom. Data represented as mean ± SD (n = 10/group).

Immunohistochemical and picrosirius red staining were utilized to evaluate differences in RTM-wrapped vs. control implants from these harvested tissues. While the skin groups had relative lower collagen density for capsule intensity in comparison to their respective muscle groups (control: 64.6 ± 9.3 vs. 70.2 ± 20.9; RTM: 60.7 ± 18.3 vs. 77.7 ± 15.2), the main effects of both the implant group (*p* = 0.76) and the tissue type (*p* = 0.07) were not statistically significant (Figure 7). However, contractile fibroblasts demonstrated changes from RTM coverage. The implant group was statistically significant (*p* = 0.03), whereas the main effect of the tissue type was not significant (*p* = 0.88) (Figure 8). Compared to the control implant groups (skin: 7.7 ± 2.6%, muscle: 8.0 ± 3.1%), the RTM groups (skin: 6.2 ± 1.8%, muscle: 5.7 ± 1.9%) had significantly fewer contractile fibroblasts, suggesting less fibrosis. Finally, TGF-β1 expression was not different. The main effects of the implant group (*p* = 0.80) and the tissue type (*p* = 0.49) were not statistically significant (Figure 9). Both groups had cells expressing TGF-β1 (~35–75% range of cells expressing TGF- β1 among groups and tissue type).

### 3.6. Evaluation of RTM-Wrapped Implants for Inflammation

**Figure 10:**
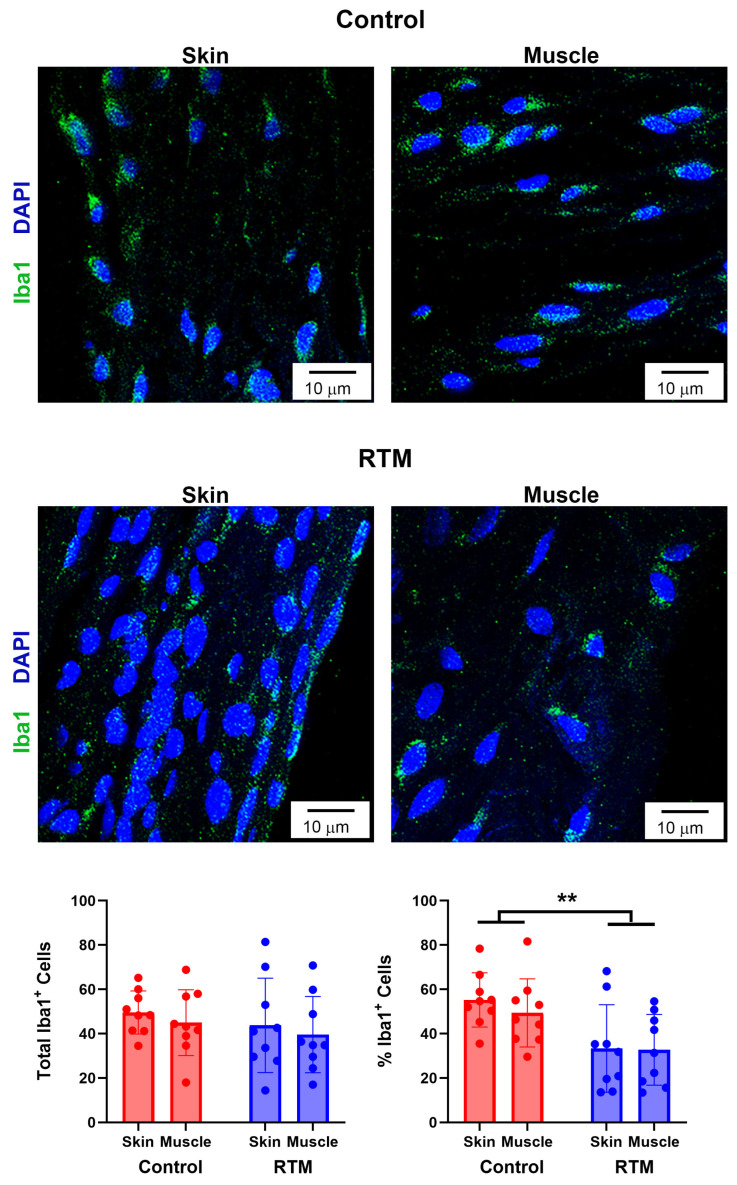
RTM coverage reduces the proportion of macrophages. Representative immunofluorescence staining for macrophages (Iba1) at 60× magnification comparing capsules from control and RTM-wrapped implants at the skin and muscle surfaces. Scale bars = 10 μm. Quantification of the percentage of Iba1-positive cells (all macrophages) are shown at the bottom. Data represented as mean ± SD (n = 10/group). ** indicates *p* < 0.01.

**Figure 11:**
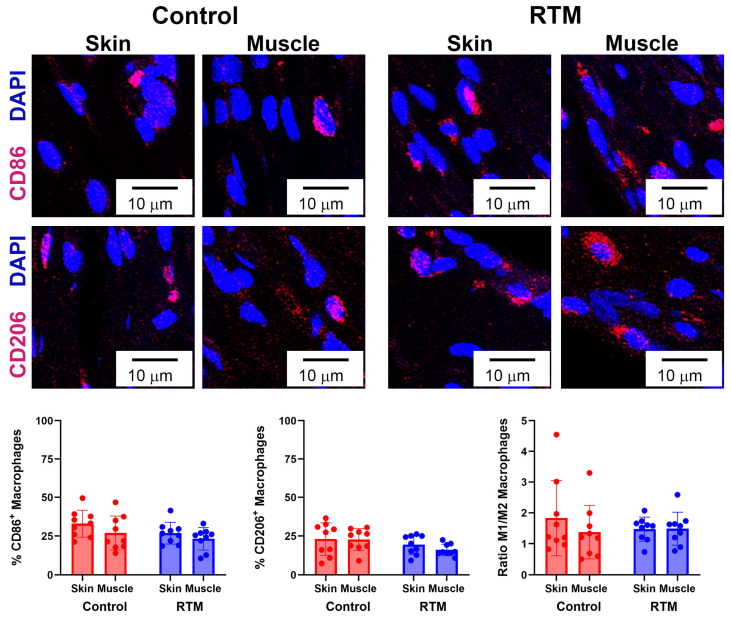
RTM coverage does not change macrophage polarization. Representative immunofluorescence staining for M1 (CD86) or M2 (CD206) macrophages at 60× magnification comparing capsules from control and RTM-wrapped implants at the skin and muscle surfaces. Scale bars = 10 μm. Quantification of the percentage of M1 and M2 macrophages are shown at the bottom. Data represented as mean ± SD (n = 10/group).

RTM-wrapped vs. control implants were also evaluated for ongoing inflammation through macrophages and macrophage polarization as part of the response to resolve a foreign body response to the implants. Analysis of the main effects showed that the RTM group had significantly fewer macrophages (*p* = 0.01) compared to the control. Tissue type (skin vs. muscle) was not different (*p* = 0.55) (Figure 10). Then, to understand the polarity of macrophages, the proportion of macrophages expressing M1 (CD86) vs. M2 (CD206) was compared. The proportion of M1 macrophages was not significantly different across the implant group (*p* = 0.10) and the tissue type (*p* = 0.10). Similarly, the proportion of M2 macrophages was not significantly different across the implant group (*p* = 0.05) and the tissue type (*p* = 0.44). Finally, examining the ratio of M1 to M2 macrophages revealed no differences in the ratio of polarized macrophages among implant groups (*p* = 0.66) or tissue types (*p* = 0.42), where both the RTM and control groups had a ratio above 1, indicative of more pro-inflammatory macrophages compared to anti-inflammatory macrophages (Figure 11).

### 3.7. Evaluation of RTM-Wrapped Implants for Neoplastic Growth

**Figure 12:**
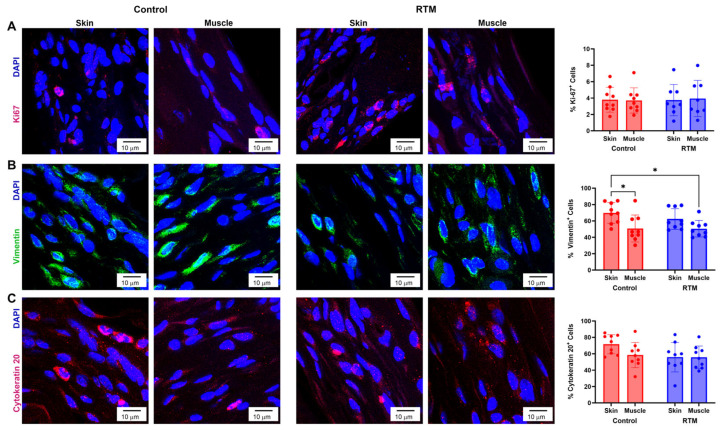
RTM coverage does not change neoplastic growth. Representative immunofluorescence staining for cell proliferation (Ki-67, (**A**)), vimentin (**B**), and cytokeratin (**C**) at 60× magnification comparing capsules from control and RTM-wrapped implants at the skin and muscle surfaces. Scale bars = 10 μm. Quantification of marker-positive cells is shown to the right. Data represented as mean ± SD (n = 10/group). * indicates *p* < 0.05.

RTM-wrapped vs. control implants were also evaluated for pathologic cellular responses during the foreign body response to the implants. The main effects of the implant group (*p* = 0.88) and the tissue type (*p* = 0.93) were not statistically significant for cell proliferation (Figure 12A). (Ki-67: *p* = 0.88; vimentin: *p* = 0.39; cytokeratin 20: *p* = 0.07) (Figure 12A). The main effect of the tissue type was significant for vimentin (*p* = 0.001), demonstrating decreased vimentin from the muscle compared to the skin (Figure 12B); post hoc tests for vimentin showed statistically significant differences when comparing the control skin group (69.7 ± 12.6%) to both the control muscle group (50.8 ± 16.5, *p* = 0.02), as well as the RTM muscle group (50.5 ± 10.1, *p* = 0.02). However, there were no differences in vimentin due to the implant group (*p* = 0.39). Finally, no changes were found in cytokeratin in the implant group (*p* = 0.07) or the tissue type (*p* = 0.19) (Figure 12C).

## 4. Discussion

Despite being one of the most common procedures worldwide, approximately half of patients who undergo breast augmentation experience complications, including capsular contracture [8]. With such a high prevalence of post-surgical complications, a better understanding of the bio-interactions between silicone implants and anatomical tissue is needed. To address this, we developed a novel model utilizing computer-aided design and 3D-printing technology to produce silicone implants that could be implanted within the prepectoral area of rats. We then utilized this model to evaluate an ovine-derived RTM used to cover the silicone implants to demonstrate the moderate advantages in foreign body response resolution compared to silicone implants alone. Mirroring current surgical trends [40], implants were placed in the prepectoral space to allow the assessment of both muscle- and skin-facing capsule characteristics. While capsule thickness did not differ between the RTM and control groups at the 12-week endpoint, we found that RTM reduced contractile fibroblasts and macrophages, suggesting that RTM may improve capsule quality, even in the absence of changes in capsule thickness.

Our study highlights the feasibility of using an accessible rodent model to evaluate RTM function. Unlike prior models where implants were placed dorsally or subcutaneously in less relevant locations, our use of a prepectoral pocket mirrors clinical anatomy. Importantly, this model theoretically allowed for precise control over implant dimensions and mechanical properties using 3D-printed molds, providing a platform for future testing of different RTM products, implant surface textures, or surgical variables. The breast implant capsules formed in this study represent a foreign body reaction that is consistent with previous studies in humans and rodent models. In humans, capsules that undergo contracture are significantly thicker than uncontracted capsules, both of which range on the order of several hundred microns [41]. Additionally, in other rodent models, lab-engineered smooth implants have also elicited capsular formation measuring several hundred microns, consistent with the results presented here [42,43]. The model presented here replicated capsular histology and size, which could aid in translatability with future treatments and strategies to mitigate common complications from breast implants such as capsular contracture. Additionally, this model could be extended to replicate capsular contracture via methods such as irradiation or the application of pro-inflammatory compounds like lipoteichoic acid [18,19,43]. The reproducibility of this model makes it an excellent starting point for the examination of a multitude of factors and variables affecting breast implant capsules, including new tissue engineering-based approaches [44].

Additionally in this study, we evaluated an ovine-derived RTM in an anatomically relevant rat model of implant-based breast reconstruction. Previous studies have demonstrated thinner capsules over ADM-covered regions [34]. In our model, average capsule thickness was not significantly different between groups; however, the RTM group demonstrated a notably narrower distribution of capsule thickness compared to control, with a smaller standard deviation and tighter range. The largest capsule in the control group was nearly twice the thickness of the largest capsule in the RTM group, while the smallest control capsule was only half as thick as the thinnest RTM capsule. This uniformity may indicate that RTM acts to stabilize the capsular response and limit remodeling, but further studies will be needed to better understand this mechanism. While speculative, these results could be interpreted to suggest that the RTM, through suppression of macrophage and contractile fibroblasts, could reduce variance in capsule thickness. ADM is thought to limit capsules via impeding inflammatory mediator interaction from adjacent tissues with the implant surface, and by effectively forming an island within the surrounding capsule, thus interfering with the centripetal contractions of the neighboring capsular tissue [13,14]. Additionally, the RTM used has been previously found to allow rapid cellular infiltration and remodeling of mature, organized vs. disorganized collagen [39,40]. Taken together, these could form a hypothesis to evaluate in future studies to understand this mechanistic aspect to reduce capsule variance.

Histologic analyses revealed that capsules surrounding RTM-covered implants had reduced contractile fibroblasts compared to the control groups. These findings were consistent with previous studies reporting reduced contractile fibroblasts in ADM-associated capsules [34]. When taken together with the reduced macrophage from RTM, these are promising findings as these methods could lead to reduced fibrosis and ultimately reduced pathological capsular contracture; however, this would require further long-term studies. Importantly, our 12-week endpoint may not be directly comparable to both human or long-term studies where the temporal dynamics of fibrosis and inflammation may not be fully captured. Specifically, the observed reduction at 12 weeks may (or may not) predict long-term clinical outcomes. Furthermore, TGFβ1 expression and collagen density were not different due to the RTM group, which tempered the effects to predicted changes in long-term fibrosis. Ultimately, more long-term studies are essential to understand these early implications regarding fibrosis.

RTM also modulated the immune response. Capsules surrounding RTM-covered implants had reduced macrophage, but they did not alter macrophage polarization. Although M2 macrophages are typically associated with tissue repair and the resolution of inflammation, and thus a desired increase from treatments, persistent M2 dominance has also been implicated in chronic fibrotic remodeling [45]. Interestingly, one prior study found after just one week that there were higher M2 levels post-implantation with ADM, suggesting that ADM may accelerate progression through the inflammatory response [37]. At our 12-week endpoint, no changes to macrophage polarization were present in the implant group, suggesting longer endpoints (i.e., 6–12 months) may be needed to understand macrophage polarization. Additionally, the use of inflammatory protein and gene panels, including cytokine expression, would better reflect macrophage polarization.

Though we observed no significant differences in collagen content by picrosirius red staining, there was a trend toward higher collagen intensity at muscle-facing surfaces compared to skin, regardless of the implant group. This suggests the possibility of regional variation in capsule characteristics, although only vimentin expression showed a significant tissue-type effect. Future studies should consider separately analyzing skin- and muscle-facing regions, as they may contribute differently to overall capsule behavior. Finally, no significant differences were observed between the RTM and control groups in neoplastic markers, including Ki-67, vimentin, and cytokeratin 20, suggesting that neither this model nor the use of RTM influenced abnormal cell proliferation.

Broader limitations for our studies are present. Although rodent models are commonly used in implant research due to their reproducibility and cost-effectiveness, they may not fully replicate the human response. Rats also heal faster than humans, potentially accelerating fibrotic remodeling and limiting the translatability of time points. Additionally, the control group did not use sham wrapping or other inert materials to understand the effects of an additional physical layer around the implant. It is unclear how significantly this would influence results, as a comparison to uncovered implants is the convention for the field. Regarding that aforementioned point, another limitation was the use of a single commercially available RTM which limited generalizability. Less has been studied regarding animal-derived RTM, but compared to human ADMs, these products provide an open and porous construct, as well as reduced elastin content; conversely, the thick and dense nature of human ADMs facilitate cellular infiltration and neovascularization. Additionally, these products came from ovine rumen, which is abundant compared to human ADM, where supply is limited by the availability of donors, as well as being typically less expensive. Future studies should explore direct comparisons between RTM and ADM, as well as longer-term outcomes in this model to assess the durability of RTM’s protective effects. Additionally, comparing different RTM sources, including human, porcine, bovine, and ovine, will help determine whether material origin influences fibrosis, inflammation, and clinical outcomes. Incorporating biomechanical testing of capsule stiffness and assessing functional endpoints may also provide deeper insight into capsule quality.

In addition, our 12-week endpoint may not capture the full-time course of capsule maturation or late complications such as capsular contracture, which can occur over several months to years. Breast implant capsules form via a foreign body reaction which begins within minutes of implantation by proceeding through the following phases: blood–material interaction, surface provisional matrix formation, acute inflammation, chronic inflammation, foreign body giant cell formation, and fibrous capsule formation and resolution. Alternatively, in capsular contracture, a pathological foreign body response occurs on an unknown time scale. Contracted capsules possess greater numbers of macrophages and T-cell infiltrate, representing pro-inflammatory signaling and further expressing greater levels of fibrosis markers compared to non-pathological capsules. At present, two main theories have been proposed to explain the development of a capsular contracture: subclinical bacterial infection/biofilm and chronic inflammation, which result in fibrosis. Although the precise pathophysiology of capsular contracture remains unknown, chronic inflammation and excessive activation of myofibroblasts could lead to capsular contracture. Therefore, long-term studies are essential to fully understand the development of this pathology. As our rat study follow-up was 90 days and was not a pathologic capsular contracture model, these limits potential conclusions regarding whether the observed changes in foreign body response would result in limiting capsular contracture. Therefore, present studies should begin to explore mechanistic understanding. Long-term studies using rat models are necessary to obtain translational conclusions to capsular contracture, especially if one considers that it is common to observe capsular contracture in humans even up to 15 years of follow-up. And finally, future studies could incorporate pro-contracture stimuli, such as irradiation or inflammatory agents to provoke pathological capsular contracture.

Limitations and further points of discussion involve the presently developed silicone-based implants. Clinical implants from manufacturers usually contain a silicone shell, first generated and then filled with silicone gel or saline. These material properties could affect mechanical signaling and biomaterial responses, such as inflammation, different than those with the currently studied implants. At present, our material and mechanical characterization of the implants is limited. As surface topography/chemistry dominates foreign body responses, further investigation of these properties would benefit this new model. Based upon previous studies, clinical implants have an elastic modulus ranging from 600 to 3000 kPa, whereas our studied implants range from ~10 to 300 kPa. The fabricated implants’ modulus values were more in line with the elasticity of the human dermis (E  <  10 kPa) and human mammary tissue (E  <  0.5 kPa) [46]. As the elastic modulus of these fabricated implants was substantially lower than commercial implants, this may have influenced the host’s response and translational relevance. Implant stiffness differences also affected macrophage polarization, fibroblast activation, and collagen tissue deposition. Mechanical forces from breast implants significantly altered the responses from cells, driving macrophages to polarize to a more pro-inflammatory state and fibroblasts to transform into myofibroblasts, leading to stiffening and collagen deposition that formed a fibrotic capsule—a key factor in capsular contracture. Human breast implants, due to their inherent stiffness, generate higher mechanical stress at the interface, which drives these cell responses more robustly than in rodents [47]. These differences highlight a key limitation to the fabricated implant designs, as these would minimize detrimental mechanical signals.

The generation of our silicone implants at a 50:1 base-to-cure ratio was congruent with previous work using silicone [48,49], therefore, there could be trace amounts of residue from the silicone curing reaction despite washing that will need additional investigation. Yet, while other manufacturers can produce miniaturized implants more akin to clinical implants, there are often scaling limits that prevent their use in murine pectoral models. Moreover, the intellectual, legal, and licensing rights in using commercial implants can be an impediment to the freedom of academic studies, or when trying additional products in combination, such as the RTM used in our studies. Additionally, while Dow Corning silicone is commonly used in murine models [50,51], clinical implants no longer use Dow Corning silicone products. Overall, these implants, while not a perfect surrogate for clinically available implants, strike a balance for reproducibility and accessibility in murine models.

Our findings support the idea that RTM may improve capsule quality as suggested by attenuating select fibrotic and inflammatory markers, even when total capsule thickness remains unchanged. In clinical terms, this could translate to softer, less symptomatic capsules and potentially reduced rates in capsular contracture. Notably, ovine-derived RTM has been shown to be more cost-effective than traditional human-derived alternatives [36]. Given that our study demonstrates similar histologic benefits with ovine RTM, namely, reduced fibrosis and inflammation marker expression, these findings suggest that ovine RTM may offer a more affordable yet equally effective alternative in implant-based reconstruction. This could be especially valuable in healthcare settings where cost constraints limit access to biologic adjuncts.

## Data Availability

Data sets are available upon request.

## Abbreviations

The following abbreviations are used in this manuscript:

RTM	Reinforced tissue matrix
SEM	Scanning electron microscopy
H&E	Hematoxylin and eosin
A-SMA	Alpha-smooth muscle actin
